# Historical roots and modern realities: Austria's sports ecosystem

**DOI:** 10.3389/fspor.2025.1498789

**Published:** 2025-03-25

**Authors:** Ivana Pranjić, Marko Begović

**Affiliations:** ^1^ Institute of European Sport Development and Leisure Studies, German Sport University Cologne, Cologne, Germany; ^2^ Faculty of Business Administration and Social Sciences, Molde University College, Molde, Norway

**Keywords:** Austria, sport, policy, sport organizations, sport funding

## Abstract

**Introduction:**

This article provides an overview of the sport structure and policies in Austria, offering a comprehensive analysis of existing frameworks and potential future developments. It explores the historical evolution of sport policy, key legislative milestones, institutional structures, and policy paradigms that have shaped the nation's approach to sports governance.

**Methods:**

The study employs document analysis as the primary research methodology, examining regulatory regimes and national policy documents. A historical outline is used to contextualize the evolution of Austrian sport policies, followed by an in-depth review of legal and regulatory frameworks at national and regional levels.

**Results:**

The analysis highlights the structural composition of the Austrian sport system, identifying key stakeholders, governing bodies, and constituents. The findings reveal the strengths and limitations of the current sport governance model, with particular attention to legislative developments and institutional interactions.

**Discussion:**

The paper discusses emerging trends in Austrian sport policy, potential areas for reform, and the role of innovative approaches in addressing contemporary challenges. The study contributes valuable insights to the discourse on sports governance in Austria and suggests pathways for future policy development.

## Introduction

The sport system in Austria is a complex and multifaceted network shaped by historical, political, and social dynamics. Understanding its structure and associated policies is crucial for evaluating the broader implications of governance and policy in the country's sporting landscape and broader societal trends. This research holds particular significance as it provides a comprehensive analysis of the historical evolution of sports policies in Austria, examining the key legislative milestones, institutional frameworks, and policy paradigms that have shaped the country's approach to sport. As the global sports environment continues to evolve, understanding the Austrian model offers valuable insights into how national and regional sports systems interact with governmental and non-governmental stakeholders, reflecting both domestic and international influences.

At the core of this study is the examination of the institutional and regulatory frameworks governing sport in Austria and the exploration of the roles of central government bodies, regional and local authorities, national sport federations, private sector entities, and other stakeholders that are interwoven within a broader governance structure that seeks to balance state involvement with private sector participation alongside a commitment to both grassroots participation and high-performance sports. The research also explores the interrelations between diverse actors, highlighting the complexities of policymaking in an environment where various interests—from public health and social inclusion to elite performance and international reputation—must be negotiated. The paper critically engages with central themes of the Austrian sport system, including the regulatory frameworks that underpin Austria's sport policies, the evolving policy paradigms in response to societal and global trends, and the interactions among key stakeholders within the Austrian sport ecosystem. The study also addresses emerging challenges and opportunities and the need for policy interventions and reforms to meet contemporary demands.

Despite a significant body of literature in understanding how the sport ecosystem has developed and related policies implemented, the data on the Austrian context is very limited (1–4). That said, the primary objective of this research is to provide a thorough examination of Austria's sport policies, tracing their historical development and analyzing current frameworks and regulatory structures that govern sport. Employing document analysis of national policy documents and regulatory regimes as the main research methodology, this study systematically evaluates legal texts, policy papers, etc., that have shaped the governance of sport in Austria. By analyzing these sources, the research aims to identify potential areas for improvement, highlight emerging trends, and suggest strategies for the integration of innovative approaches to address contemporary issues within the Austrian sports system.

Ultimately, the study seeks to contribute to the broader discourse on sport policy and governance, offering a nuanced understanding of Austria's approach to sport and its potential for future development. The research represents a pioneering effort to offer a comprehensive understanding of the sport ecosystem in Austria, especially in terms of work dedicated to understanding the macro level of sport in the country, its governance, and the policies that shape its developments. Existing research in this field has often been fragmented, with a predominant focus on specific aspects of sport, such as winter sports and tourism, and the societal influence of certain sports, mainly soccer and skiing. Consequently, there is a notable lack of integrative studies that offer a comprehensive analysis of the Austrian sport ecosystem, encompassing the historical, political, institutional, and sociocultural factors that shape the country's sports policies.

This gap in academic literature underscores the need for comprehensive analysis examining legal and regulatory frameworks and the broader socio-historical and political dynamics that influence policy development. Such analysis is essential for understanding the roles and interactions of key stakeholders and how their collective actions shape the national and regional sport landscape. By synthesizing existing knowledge and contributing new insights, this study aims to fill this research gap, offering a critical contribution to the field of sport policy and governance in Austria.

## The sport system in Austria

The sport ecosystem in the Federal Republic of Austria is shaped, to a large extent, by the federalized structures. Divided into state, non-state, and mixed entities, the sport structure in Austria features both public sport administration and the structures of autonomous civic or self-administration of sport, i.e., the non-governmental sport sector and sports-related organizations.

Sport is an important part of life for many Austrians: 73% engage in sport at least once a week. The most popular (grassroots) sports are cycling and swimming, regularly practiced by 38% and 23% of the population, respectively. Approximately 15,000 sport clubs are registered in Austria. By the end of 2022, there were approximately 2,020 football clubs in Austria, making football the sport with the greatest number of clubs, followed by tennis with approximately 1,680 clubs and equestrian sports with approximately 1,430. The sport with the highest number of members in sport clubs in Austria is football, with approximately 297,000 organized athletes, followed by tennis with approximately 194,000, and skiing with approximately 133,000. Besides sport clubs, fitness centers/gyms also play a significant role in the Austrian sport market. In 2020, there were 578 fitness studios in Austria, the highest number ever recorded (5).

Historically, sport in Austria has not been prominently supported as a part of the educational system, resulting in a broader operational setting—the sport clubs setup (6). From an academic perspective, sport is also under-researched in Austria (7). The positive effects of sport on state goals are being realized through federal state efforts. On the national level, a model of policymaking (inclusive of diplomacy through sports) includes the use of sport success to strengthen the soft power of the state, the involvement of athletes in politics and diplomatic processes, using sport as a diplomatic instrument through international development programs, and the organization of major sport events and less well-known but traditional international sport events. Although the benefits of sport for Austria are generally (partially) recognized, it has not yet been strategically designed (8, 9). Sporting success, especially in winter sports, represents an important economic driver for the country (10). As sport is becoming a highly relevant foreign policy tool (11), it is suggested to give higher relevance to the support for the construction of a quality and sustainable national sport system. An interdepartmental development of a national sport strategy is recommended or separate programs with similar goals.

## Literature review

Understanding the political and policy aspects of sport has not primarily been on the research agenda in Central Europe (12). Academic literature on sport policy and governance in Austria is very limited and remains an under-explored area within the broader field of sport studies. A significant body of work examines the historical roots of sport in Austria (6, 13–15). Müller and Weiß (16) can be considered the main authors researching the historical and sociological perspectives of sport in Austria. While Müllner (17) focuses his research on the historical aspects of sport, Weiß's contributions take a sociological (socio-historical) lens, with the main research focus being sociology in sport (18), societal developments and implications for health, and grassroots sports participation (19, 20). Much of the existing literature focuses on certain aspects of the sport system, mainly (winter) sport and tourism and football and skiing as elements of community engagement (14, 21). Gender equality, a major issue in leadership in sport governance today, has been the focus only from a historical perspective by Dorer and Marschik (22). Schnitzer et al. (7) wrote about the (potential) strategic approach of youth multi-sport events in Vorarlberg and Montafon (EYOF^1^ Innsbruck 2015, YOG^2^ Innsbruck 2016, ICG^3^ 2016). Social inclusion and disability in sports were reviewed by Wojciechowski and Stura (23).

The economic dimension of sport has been academically considered by Barth et al. (24). On behalf of the responsible Federal Ministry^4^, a so-called “Sports Satellite Account” is published periodically. The latest report was published in 2019 (25).

Though a dozen studies on sport, history, and sociology in Austria exist, none of the studies focus on sport politics and policy in Austria. One of the few academic contributions on this topic is authored by Weiß and Norden (6), discussing the relevance of sport clubs in Austria. That said, these individual contributions lack the sort of synthesis to gain a better understanding of the sports ecosystem as a whole.

The Austrian political system rests primarily on the interplay between two major political parties within both the central and subnational levels as part of the prolonged democratization process of the country. The development of policies, including sport-related ones, requires a process that includes submission before the *Nationalrat* (National Council or the parliament), which is in charge of adopting the final draft of legislative and policy documents. Prior to this, and depending on their nature, the policies are prepared by the competent federal ministry and adopted by the government. The preparation phase includes the draft version being sent to subnational authorities for review processes. Nominally, the coordination between central and subnational authorities is based on a social partnership shaped by the significant level of the autonomy of the latter, reflecting the need to balance heterogeneous policy networks (26). It implements a form of institutional corporatism as part of the ordinary process of directing various interests from different stakeholders that outputs policy formulation and adoption (27). That said, the policy processes have proven to be challenging as a multisectoral approach with coordination at both the horizontal and vertical levels of the public sector and with non-governmental and other stakeholders is required. Together with corporatization, politicization and governmentalization represent the dominant drivers behind these processes. That said, the policy processes unfold as the result of the interplay between different stakeholders and their ideas in politically shaped institutions through the framework of multi-level governance. Researching multi-governance is not a new phenomenon, and the practice depends greatly on the socio-political realm and democratic capacity. In Canada, the system rests on the decentralization of competences to meet regional-specific needs, while in Bosnia and Herzegovina, the exercised model led to fractured governance with subnational units acting more in the form of independent actors (12, 28). Moreover, with respect to these differences, in Canada, the focus has been on accountability as an importance governance dimension, including the role of the regional policy within national sport policy, while in Bosnia and Herzegovina, the primary priority of the regional actors is maintaining a monopoly within its borders despite this often leading to friction, conflicting policies, and facilitating corrupt practices (29). Similar to the Canadian example, despite the fact of strong subnational actors in Germany, the level of coordination maintains consistency in policymaking (30).

However, given the fact that regulations in sport ecosystems are quite heterogeneous and equally diverse, the case of Austria should be observed independently. Furthermore, in the context of comparative sport policy analysis, the example of Austria has not been researched in any of the big handbooks on comparative sport policy and politics (31–33). Emerging trends in the Austrian sport sector, including innovations in governance, digitalization, inclusivity, and gender equality, have not garnered attention in recent literature at all. There has not been any study addressing sport governance and sport policy and politics in Austria since 2015. These trends point to the need for policy frameworks that can adapt to contemporary challenges and opportunities in the sporting landscape.

## Methodological considerations

Given the research inquiry, the interplay between different levels of public sectors will be examined. As Austria is a federal state, the institutional relationship between these actors and their relationship with the sports movement is the main interest. With the limited data on the Austrian sport ecosystem in mind, the research focus is on document analysis to extract, explain, and understand the dynamics and implications (34).

In particular, formal regulatory regimes (legislation) and policy documents represent the major sources for this inquiry. In the first step, academic publications concerning the utilization of sports in Austria were gathered. Second, publicly available governmental policy and legal documents (from each ministerial department) were collected and analyzed. To identify relevant academic publications and policy documents, we applied the following keywords in both English and German: sport policy in Austria and good governance in sport in Austria. Third, relevant online media archives and articles between 2010 and 2020 were screened.

The collected documents were organized chronologically, including strategies and action plans, academic papers, websites, and policy documents related to the ministries and their programs. The scarce amount of relevant academic literature and policy documents posed considerable challenges for collecting sufficient empirical data. Table 1^5^ outlines the legal documents, sport acts on national and regional levels, and regional and national sport strategies we used as primary sources. Moreover, they were clustered and further analyzed depending on their nature and relation to the field of sport. Under the patronage of Sport Austria, the *Bundessportmagazin* (Federal Sports Magazine) serves as a key source of information, offering reports and analyses on a range of topics, including sport strategy, sport medicine, grassroots sports, sport organizations, innovations in sport, and integration within the sport sector. It is distinguished as the sole print media outlet in Austria, providing coverage of sport policy issues. Legislative framework, regulatory regimes, and related policies represent major sources for this analysis. These documents were clustered in relation to the competent institution in charge, especially regarding adopted laws, strategies, and other regulations. Furthermore, employing critical policy analysis should provide a better understanding of the level of coordination and consistency in the formulation, development, and operation of the sports ecosystem and related policies.

**Table 1:** Data sources for the applied document and policy analyses.

Source of data	Austria
Academic publications	Bruckmüller (13)
	Iber et al. (35)
	Horak and Spitaler (21)
	Müllner (17)
	Müller and Weiß (16)
	Schnitzer et al. (7)
	Weiß (83)
	Weiß and Norden (18)
	Weiß et al. (19, 20)
Government policy documents	• National Action Plan for Physical Activity • Austrian 10 Health Targets • *Nationaler Aktionsplan Behinderung* 2022–2030 • Masterplan Cycling 2015–2025 Strategy • Masterplan Walking • Health Strategy for Children and Young People - *Regierungsprogramm* 2020–2024 - Carinthia Sports Act 1997 (KLGBl 99) - Upper Austria Sport Act (OÖLGBl 1997/93) - Salzburg State Sports Act 1988 (SLGBl 98) - Styria State Sports Act 1988 (StLGBl 67) - Tyrol Sports Promotion Act (TLGBl 2006/97) - Vorarlberg Sports Act (VLGBl 1972/15) - Vienna State Sports Act (WLGBl 1972/17). - Lower Austria Sport Act (NÖLGBl 5710-0) - Federal law on the reorganization of federal sports facilities - Associations Act - Federal Law on Anti-Doping - National Sports Promotion Act 2017 - Federal Gambling Act - Federal Ministries Act 1986 (updated 7.5.2023) - Sport *Steiermark* (2014). *Sportstrategie* 2025. *Referat* Sport. - Sport.Wien.2030. (2020). *Sportstätten-Entwicklungsplan/Herausgeber Stadt Wien-Sport Wien. Wien: Stadt Wien-Sport Wien.* - *Sportstrategie* 2030 *Stadt Graz* (2019). - Styrian Sport Facilities Protection Act 1991. - Vienna Sports Promotion Contribution Act
Relevant online media	*Bundessportmagazi*n https://www.bundessportmagazin.at/

Source: compilation by authors.

Data analysis was conducted using a critical policy approach, providing a structured approach to examine the institutions, ideologies, key actors, administrative structures, and contextual factors (36). This approach was informed by broader institutional theory (37), allowing for a comprehensive examination of the institutional structures and dynamics influencing policy development. The data was coded deductively, guided by the core constructs of critical policy analysis. During the initial coding phase, these constructs were systematically applied to the dataset. Following this initial coding phase, related codes were categorized into broader themes, which were then further refined into higher-order themes. The key themes of the critical policy analysis that were identified were policy-driven public sector governance led by financial and funding support for Austrian sport, including decentralized governance structures and autonomous sport organizations as stakeholders operating under a framework of self-regulation.

## Tracing the historic arc of sports in Austria

As a democratic and federal republic, Austria is known for its cultural heritage throughout history, from architecture, literature, and music to theater and film. The “modern Homo Austriacus” sports identity started with skiing after the Second World War (38). In 1862, the first sports clubs for rowing and mountain climbing were founded (38, p. 30). Preceded by the proclamation of the constitution of the Austrian empire by Franz Joseph I and the liberalization of the club policies in 1861, the permanent founding of sports clubs took place, followed by the founding of sport federations beginning in the 1880s (38). To date, sport is of high relevance for Austrian society, conveying important values of social interaction and coexistence, ranging from health-related to elite-level practice. As noted in Article § 1 of the Austrian Sport Promotion Act 2017 [“*Bundessport-förderungsgesetz* 2017” (BSFG 2017)], the relevance of sport for Austrian society is understood through the promotion of the values of bringing together individuals from diverse cultures and social backgrounds, bridging generations, fostering health, unity, integration, communication, solidarity, and enthusiasm for a common purpose. Therefore, it also represents a crucial public interest today. Aiming to overcome the capitalistic order and differentiations of the sport clubs, three umbrella sport organizations that exist to date—the Austrian Association for Sport and Physical Culture [*Arbeitsgemeinschaft für Sport und Körperkultur Österreich* (ASKÖ)], the Austrian Sports Union (SPORTUNION), and the Austrian General Sports Association [*Allgemeiner Sportverband Österreichs* (ASVÖ)]—were established.

In the midst of the Second World War, there was already a “call to all Austrian athletes!”. A “Central Office for the Reestablishment of Austrian Sports” was intended to establish a communist-dominated standardization of sports in Austria with the help of the Soviet army leadership. In May 1945, a working committee meeting in the State Secretariat for Popular Enlightenment, Education, and Cultural Affairs met with representatives of the gymnastics and athletics union, aiming to bring together athletes from all disciplines into a unified group to develop a large Austrian sport movement. Due to difficulties that arose in the preparation and implementation of this event, the unified Austrian sports movement failed. The Workers’ Association for Sport and Physical Culture (since 1971, “ASKÖ”) and the football association rejected the Central Office for the Reestablishment of Austrian Sports. The planned central office was perceived as a communist organization that would disregard the ideals of workers’ sports (39). toward the end of 1945, the Central Office for the Reestablishment of Austrian Sport was taken over by the reactivated Austrian Main Association for Physical Sport (Ger.: *Österreichischer Hauptverband für Körpersport*) (ÖHVfK) (38). Both the Central Office for the Reestablishment of Austrian Sports and the Austrian Main Association for Physical Sport did not significantly influence sporting events in Austria after 1945 and increasingly lost importance after the founding of the Austrian Olympic Committee in December 1946 and the establishment of ASVÖ in 1949.

Since the end of the 19th century and the beginning of the 20th century, Austria's sport have evolved in two directions. On one side, there were performance-oriented associations for individual sports, and on the other side, ideologically or denominationally oriented sports organizations emerged. From the ruins of the Second World War, the Second Republic was established, alongside a reorganization of sport in Austria. Alongside the politically oriented umbrella organizations ASKÖ and SPORTUNION, the desire for a non-partisan, independent sport umbrella association became unmistakable. Organized in a federalist manner, ASVÖ soon gained the respect of independent sport federations and established itself as a significant entity in Austrian sport.

## Sport structure in Austria

The legal ambiguity of sport within the public sector starts with the definition of sport. The EU Sport Charter defines sport as “all forms of physical activity which, through casual or organized participation, aim at expressing or improving physical fitness and mental well-being, forming social relationships or obtaining results in competition at all levels” (40). While the currently in act Austrian Sport Promotion Act 2017 (BSFG 2017) lacks a definition for “sport,” it does provide definitions for “grassroots sport” and “elite/high-performance sport” (41). As the Federal Constitution does not mention sports in any way, the general clause of Article 15 B-VG applies, transferring competences in legislation and enforcement in sports matters to the nine federal states, ensuring high juridical and administrative autonomy. To ensure a hierarchical concept of self-governance, sports in Austria are organized on a club membership basis (42). The Associations Act (BGBl. Nr. 233/1951 from 28 August 1951 to 4 April 1962; BGBl. Nr. 102/1962, *Vereinsgesetz* 2002) (43) builds the legal basis to establish a club. The Act contains precise provisions on founding and business operations in the field of sports. A club is defined as any voluntary and permanent organized association of several people to achieve a specific common purpose through continued common activity (44). Another important law is the Federal Law on Anti-Doping 2007 [Anti-Doping-*Bundesgesetz* 2007 (ADBG 2007)] (45).

The federal state level exercises a high level of autonomy in practice. This is shown by four out of nine federal states (Vorarlberg, Lower Austria, Upper Austria, and Tirol) having a federal sports strategy, along with three cities (Graz, Dornbirn, and Salzburg) adopting a main policy document. The capital city and the federal state do not have a sport strategy; instead, a sport facilities development plan, “*Sport.Wien.2030*”, was developed (46). The funding competence of the federal government (“private sector administration”) refers to Article 17 of the Federal Constitution: “The position of the federal and state governments as holders of private rights is not affected in any way by the provisions of Articles 10 to 15 regarding competence in legislation and enforcement” (Article 17 B-VG). Furthermore, the Federal Sport Promotion Act (41) (“*Bundessportförderungsgesetz*”), the Federal Gambling Act (47) (“*Glücksspielgesetz*”, GSpG), and the Associations Act (43) (“*Vereinsgesetz*”) regulate sport through the law. From 1 January 2018, the BSFG (81), together with the Federal Gambling Act, built the legal basis for the federal funding of sport. The Federal Sport Promotion Act 2017 established the Bundes-Sport GmbH (BSG) (Federal State Ltd.) as a subsidiary funding agency, 100% owned by the Federal State.

The promotion of physical activity in Austria is coordinated at the national level through a collaborative mechanism involving key government bodies. Specifically, the Austrian Federal Ministry for Arts, Culture, the Civil Service and Sport, in conjunction with the Austrian Federal Ministry for Climate Action, Environment, Energy, Mobility, Innovation and Technology, plays a pivotal role in this endeavor. These ministries co-lead the Working Group on Health Target Number 8, which was established in 2014. The primary objective of this working group is to foster healthy and safe exercise and physical activity in daily life by creating and sustaining appropriate environments. This interministerial cooperation underscores the government's commitment to enhancing public health through the promotion of physical activity and the creation of supportive infrastructures.

The Austrian 10 Health Targets initiative aims to enhance the overall health of the population, including individuals with disabilities, with the specific objective of extending the average healthy life expectancy by 2 years over the next two decades. Within this framework, the promotion of physical activity is a critical component. The initiative seeks to integrate adequate levels of physical exercise into daily routines by improving residential environments and infrastructure. This includes the development and maintenance of cycle paths, playgrounds, recreation rooms, and active commuting options for educational institutions. The target groups for these interventions encompass kindergartens, schools, nursing homes, and various community clubs. By fostering environments that facilitate and encourage physical activity, the initiative aims to embed healthy habits into the daily lives of individuals across all age groups and societal segments.

Some federal states emphasize the relevance of sport in all its forms or highlight the significant importance of sport in people's lives and society [Lower Austria state government, 2020, §1 (48); Upper Austria state government, 2019 (49)] and emphasize the important role of sport in maintaining health, moral and physical education, and international understanding [Lower Austria state government, 2020, §1, Salzburg State government, 2018 (50)]. Only the Vorarlberg Sport Act offers a definition of sport, with sport being understood as a physical activity intended for recreation or exercise [Vorarlberg state government, 2022, § 2 (51)]. Excluded from this definition of the BSFG are mandatory and voluntary school sport in Austria and all forms of sport not organized in clubs, schools, etc., yet pursued for similar reasons.

## Sport policymaking

Sport policymaking in Austria is decentralized and, as such, a matter of the federal states and organizations in clubs/associations guaranteeing the concept of autonomy in sport (24). The foundation for the decentralized sport ecosystem within the public sector lies in sharing responsibilities between institutions at all levels, i.e., the national and subnational (regional and local) levels, following the principle of subsidiarity. The sport systems across the Pan-EU level are characterized as heterogenous, from the operational perspective, according to Andre–Noel Chaker's study “Good Governance in Sport, a European Survey,” and based on the two models (interventionist and non-interventionist), access to the legal regulation of sport can vary due to how responsibilities are distributed, whether state competence centralized or decentralized, and whether there is a consolidated or unconsolidated system in place (52). The two models mentioned are interventionist and non-interventionist and can also be called autonomous models (53). In this European study from 2004, Austria's sports legislation was categorized as non-interventionist, focusing on the regulation of financial and logistical aspects of support for sport. In contrast, the interventionist model catalyzes legislative structures for sport (52).

According to Article 15 of the Constitution (B-VG), sport is the responsibility of the federal states and a competence of the nine federal states, with each having an appointed regional director for sport (nine in total). This has been the case since the adoption of the federal constitution in 1920, according to which only those matters that are explicitly listed are federal matters, and sports were not listed (54). In 1946, on the occasion of the “Week of Sport: 950 years of Austria”, Austria presented itself for the first time as a unified entity to the public, as all sport federations participated in the event (55).

Based on Article 17 of the Federal Constitutional Law (“private sector administration”), the federal government primarily performs a funding function. The reference to sport in the constitution is mainly associated with the power dynamics between national and federal/regional governments rather than the constitutional protection of sport as an activity of public interest. The two main laws referring to sport on the national level are the Federal Law on Anti-Doping 2007 [(Anti-Doping-Bundesgesetz 2021 (ADBG 2021)] and the BSFG (2017). The BSFG (2017) converted the Federal Sport Promotion Fund *ex lege* into the BundesSport Ltd., whose shares are 100% owned by the federal government, a legal entity under the public law, obliging the responsible minister to define strategic priorities for national funding (BSFG 2017 § 7 p. 4 and § 10 p. 4) (41), aiming to improve the efficient performance of potential-oriented funding processes, strengthen and professionalize the associations, and secure and further develop knowledge and information management. Since 2023, the statutory minimum funding amount equals 120 million Euros according to the GSpG (47), with 50% for competitive and elite sport, 45% allocated to grassroots sports, and 5% to support Austrian-wide organizations with special tasks in sport.

Strengthening and supporting anti-doping work is a key goal of the BSFG (2017). According to the Federal Anti-Doping Act (2007) and in accordance with the UNESCO Convention against Doping in Sport (2005), the Austrian National Anti-Doping Agency (NADA) was established, regulating cooperation with the independent Austrian Anti-Doping Law Commission (ÖADR) and the Independent Arbitration Commission (USK). This legislation recognized NADO as a private limited company, including specific provisions within the Austrian Medicines Law (56). In addition, NADA Austria is responsible for deciding on applications for medical exemptions (TUE), reporting on compliance with anti-doping regulations by Austrian sport organizations, and outlining NADA Austria's cooperation with government investigative bodies.

The foundation of civil organized sport in Austria are the clubs, both on the competitive and elite and on the grassroots levels. These implement the sport portfolio and join forces regionally at the federal state level and on a national level via the three national sport umbrella organizations and national sport federations. The three main organizations and state institutions also act and operate on the federal and state levels, with sport entities on the one hand and those 100% owned by the state but outsourced as limited liability companies and non-governmental associations (associations, clubs, and institutions) on the other hand. On the national state level, the federal state institutions, mainly the different ministries, are in charge of various areas of sports. As described above, according to Article 15 of the Constitution (B-VG), sport is the responsibility of the federal states and a competence of the nine regions. Listed as a key aspect of the government program 2020–2024, the professionalization of the sport associations with the goal to establish a Professional Sport Law that improves the framework conditions for sports-specific professions in labor, tax, and social security law through the recognition of the specificities of sport and the elimination of existing inequalities is foreseen. Moreover, by strengthening the autonomy of organized sport via performance agreements through multi-year funding under the coordination and leadership of the Austrian Federal Sport Organization Sport Austria, the valorization of voluntary commitment, advancement of “green sport,” support professional development of sport staff and gender equality, etc., have gained relevance in governmental spheres.

On the national and state levels, six ministries are entrusted with sports: (a) Federal Ministry for Art, Culture, Public Service and Sport, (b) Federal Ministry of Defense, (c) Federal Ministry of Education, Science and Research, (d) Federal Ministry of Internal Affairs, (e) Federal Ministry of Finance, and (f) Federal Ministry for Social Affairs, Health, Care and consumer protection. Moreover, there are the nine federal state governments with nine state sports organizations (LSO) and the BSG. On the federal state level, sport is part of the “Sport division” of the Federal Ministry for Art, Culture, Public Service and Sport. The five divisions located within the ministry manage matters related to “Sport strategy, international, legal, top sport & professional youth sport and grassroots sport.” Army sport is the responsibility of the Federal Ministry of National Defense. School, university, and academy sport are in the responsibility of the Federal Ministry for Education, Science and Research. Health promotion and prevention are the responsibility of the Federal Ministry for Social Affairs, Health, Care and Consumer Protection. Finally, the Federal Ministry of the Interior and the Federal Ministry for Finance have a section dedicated to sports-related matters (47).

Cooperation and collaboration between the institutions are content-related. The “National Action Plan for Physical Activity”, for example, was developed by cooperation between the Federal Ministry of Interior and the Federal Ministry of Education, Science and Research.

The inaugural National Action Plan on Physical Activity (*Nationaler Aktionsplan Bewegung*) was developed collaboratively by the Federal Ministry for Arts, Culture, the Civil Service and Sport and the Federal Ministry of Social Affairs, Health, Care and Consumer Protection. This comprehensive plan, adopted in April 2013, was developed in accordance with the EU Physical Activity Guidelines, promoting a cross-sectoral approach (health in all policies), aiming to implement cross-sectoral measures designed to significantly enhance public awareness regarding the importance of physical activity. Furthermore, it seeks to positively influence the physical activity behaviors of the population in alignment with national physical activity recommendations. Through coordinated efforts across various sectors, the plan endeavors to foster a societal shift toward more active and healthier lifestyles (57).

The competences for financial funding of school sport organizations (School Olympics, etc.) are the responsibilities of the Federal Ministry for Art, Culture, Public Service and Sport and the Federal Ministry of Education, Science and Research. Although, formally, youth sport (D4) is part of the agenda of the Federal Ministry for Art, Culture, Public Service and Sport, basic competences and policy development are realized on the subnational level.

In the public sector, there are several companies dealing with sport. These are the NADA, Austrian Institute for School and Sport Facilities, Federal Sport Facilities Company, Austrian SportAid Foundation, Austrian Institute for Sport Medicine, and the Institute for Med. and Sport Scientific Consultancy, each with a different legal status. Some institutions and organizations are limited liability companies owned by the state or federal states with a fixed percentage distribution; others are NGOs with competences from the state. Furthermore*,* 100% Sport is the Austrian Center for Gender Competence and Safe Sport. Set up by the Ministry of Sport, it is an autonomous association to promote gender equality and safe sport agendas in Austrian sports. The Association for the Preservation of Integrity in Sports is the ombudsman's office of the Play Fair Code (PFC), which aims to guarantee clean and manipulation-free tournaments in cooperation with Austria's sport federations.

The Austrian SportAid Foundation is a relevant factor in financing top-class sports. Constituted as an association, it pursues the goal of making top-class Austrian sport internationally competitive by creating the necessary economic conditions. Financial resources are provided from projects with business partners and the public. The federal sport facilities were outsourced from the federal administration in 1999. The ownership of the land and facilities was transferred to the Bundessporteinrichtungs-GmbH (BSPEG). With the establishment of the BSFG in 2017, the BSPEG became a subsidiary of the BSFG (81). The main objectives of the BSPEG are the management, maintenance, and improvement of the sports facilities created by the federal government while promoting top-class and competitive sports and training and continuing education offerings, in particular in schools, educational institutions, and universities, and promoting grassroots sports. The Austrian Sport Resorts operate several federal sports and leisure centers (BSFZ) (58).

**Figure 1:**
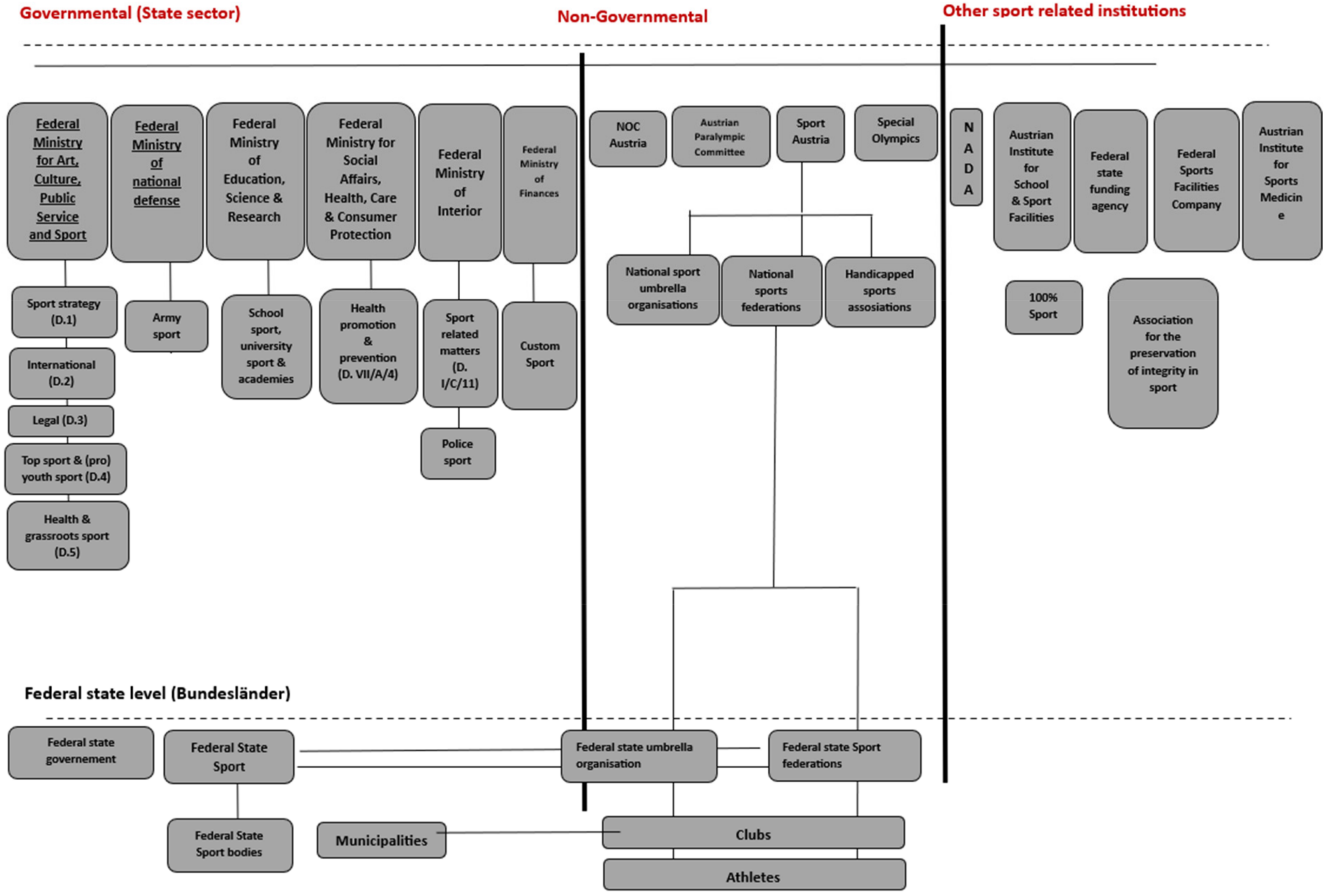
Austrian sports system. Source: authors’ representation.

On the federal state level, federal state sport organizations (LSOs) are defined as corporations under public law, mainly the respective federal state sports law (as described in Article 15 of the Constitution), based on the distribution of constitutional competences/responsibilities, resulting in nine different federal state laws from the nine federal states. The legal basis is the respective federal state sports laws, which also define the scope of duties (Figure 1).

The responsibility for sport lies with the Federal Ministry for Art, Culture, Public Service and Sport. The task of the Sport Section (“*Sektion Sport*”) is to promote the function of societal, social, and health policy in sports, sports clubs, and associations. The Federal Ministry for Art, Culture, Public Service and Sport is primarily responsible for the state sector, and Sport Austria (a federal state organization) assumes this responsibility for the non-state sector.

Since 2000, the positioning of sport on the ministerial level has undergone some organizational changes in Austria. With each governmental term, the responsibility for sport was moved to different ministries, from the federal chancellery (2003–2009) to the Federal Ministry of Defense and Sport (2009–2018) and to the Federal Ministry for Public Service and Sport (BMöDS) (2018–2020). Since 2020, sport affairs have been handled—as indicated in Figure 1—by the Federal Ministry for Arts, Culture, Public Service and Sport (BMKÖS), reflecting the changing prioritization and integration of the sport sector within the Austrian federal government.

The frequent reconstruction of the Ministry of Sport in Austria indicates that the sport sector has not maintained a stable, independent institutional presence within the government. Instead, it has repeatedly been reassigned or incorporated into other ministerial portfolios, potentially reflecting several underlying factors. The integration of sport into larger ministries rather than maintaining a dedicated ministry suggests that it is often perceived as a secondary policy area, highlighting its low political prioritization. As a result, sport governance has often been subject to shifting governmental structures rather than being recognized as a core policy pillar.

Governments have sought to streamline ministerial operations by consolidating responsibilities, which has led to sport being placed within different departments depending on broader governance strategies. However, frequent reassignments may have led to inefficiencies, inconsistencies in policymaking, and challenges in long-term strategic planning for the sector.

Political dynamics have also influenced these changes, as shifts in government leadership follow different political agendas and the repositioning of sports governance. The lack of continuity in the placement of sport governance suggests that it has been more of a flexible policy area rather than a core consistently managed domain within Austrian politics.

Finally, the absence of a long-term strategic framework for sport policy in Austria is evident in these repeated shifts. Without a stable institutional structure, challenges in the implementation of coherent policies to secure sustained funding and foster long-term development programs arise. This lack of consistency may hinder Austria's ability to develop a robust sport policy framework, affecting not only elite sports but also grassroots initiatives, public health strategies, and international collaboration.

## Non-governmental sports sector

In the non-governmental sector, Sport Austria [formerly *Bundessportorganisation* (BSO), Eng.: Federal Sport Organization] is the umbrella organization of Austrian sport, legally a private law association, coordinating sport matters with the responsible state bodies. Above all, Sport Austria is a link between state institutions and sport organizations in Austria. According to the statutes, the main purpose is the dissemination and promotion of sport and the protection and representation of the interests of sport within and outside of Austria (59). The full members of Sport Austria are the three national (grassroots) umbrella organizations—ASKÖ, ASVÖ, and SPORTUNION—the 60 recognized national sport associations, the Austrian Disabled Sport Association, the Austrian Olympic Committee, the Austrian Paralympic Committee, and Special Olympics Austria (59, § 6 s 3). The extraordinary members are the Austrian associations of significant importance to Austrian sports (e.g., Austrian Company Sport Association and Austrian Army Sport Association), such as funding bodies for Austrian sport, sport science or sport medicine institutes, local authorities responsible for regulating and promoting sport in Austria, the federal state, or other organizations relevant to sport in Austria (38).

Voting rights for different bodies and working groups and the general assembly at Sport Austria are given only to full members in accordance with the statutes (38). This is particularly important for practicing self-governance as in 2020, Sport Austria initiated and developed the “Good Governance Code of the Austrian Sport.” The Code aims to provide members of Sport Austria with guidelines and a set of rules for the organizational structure, government bodies, and monitoring systems (60). However, the Code is rather a non-binding set of rules, and it 's up to the members to adhere to it. The manner in which sports have been regulated in Austria is historically influenced. The three national (grassroots) umbrella organizations—ASKÖ, ASVÖ, and SPORTUNION—are specific to the Austrian sport system. Nearly all clubs are members of one of the three because of reasons based on the political history of the country. SPORTUNION emerged from the Christian gymnastics movement, ASKÖ continues the workers’ sport movement, and ASVÖ claims to be politically independent.

The origin of ASKÖ can be traced back to the Austrian Workers Gymnastics Association (*Österreichischer Arbeiter-Turnerbund*), founded in 1910, which partnered with workers’ gymnastics clubs close to the Social Democratic Party in the early 1890s. Cycling, hiking, swimming, and athletic clubs followed. Worker's sport and gymnastics clubs, together with the soldiers’ sport organizations, united to form the Association of Workers and Soldiers Sports Clubs (*Verband der Arbeiter- und Soldatensportvereinigungen*), which then grew into ASVÖ. ASKÖs ideals of sport were solidarity, teamwork, and healthy bodies. Sport was part of the workers’ lifestyle and aimed at the masses (6, 38). ASKÖ was banned from 1934 until the end of the Second World War and was newly founded in 1945, building on its traditions from before the ban in 1934.

Records of the very first steps toward founding SPORTUNION are sparse. A failed attempt at a unified Austrian sports movement and the division of Austria into four occupation zones made organic interactions impossible. Each federal state relied on its own design of a sports organization within the federal state, meaning that the historical and organizational development within SPORTUNION took place independently of each other in all federal states (39). The national organization SPORTUNION was established on 2 May 1945 and was an association based on Christian values uniting gymnastics and sport in Austria and—according to their statutes—independent of parties. Organized on a federal basis, 26 June 1949, ASVÖ was founded in Vienna as an impartial and independent association and counterpart to the already established (party political) ASKÖ and SPORTUNION, intended to offer “a home” to all those—different in ideology—as the two other associations (61).

**Figure 2:**
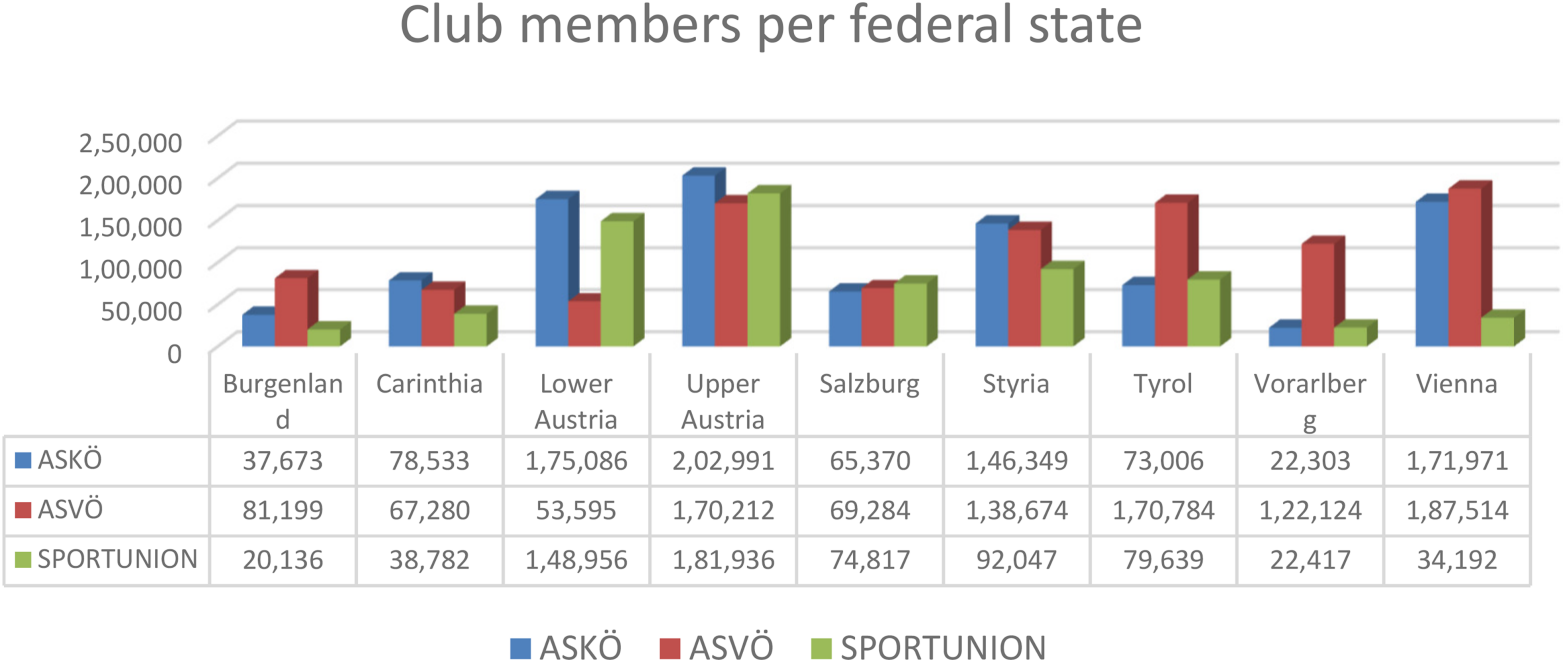
Club members per federal state. Source: Sport Austria (82).

**Figure 3:**
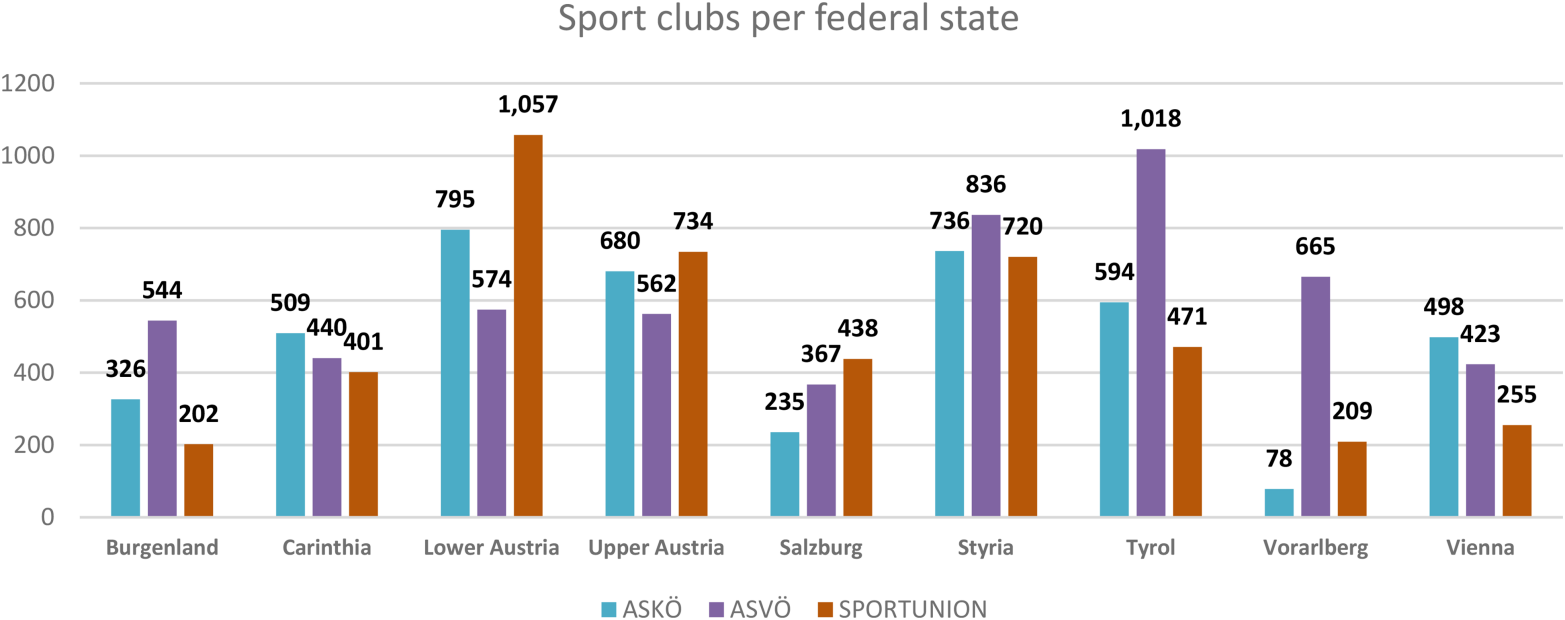
Sports clubs per federal state. Source: Sport Austria (82).

Based on the Sport Austria member survey 2023, as of 31 December 2022, ASKÖ, ASVÖ, and SPORTUNION had a total of 2,726,870 registered members (Figure 2). With 1,060,666 registered members, ASVÖ had the highest member rate of the three national (grassroots) umbrella organizations. ASKÖ had 973,282 members, and SPORTUNION had 692,922 registered members (Figure 3) (82). More than half a million people engage voluntarily in sports clubs (62).

A comparison with the micro census of the National Institute for Statistics “Statistik Austria” on club membership numbers shows divergence. Sport Austria numbers come directly from the three head entities, SPORTUNION, ASKÖ, and ASVÖ, with estimated double counts and inactive members (parents of kids; athletes are multi-members), whereas Statistik Austria determined active members.

It is essential to address the issue of unequal treatment of various sports disciplines in statistical analyses. The disparities in data collection and interpretation can lead to skewed representations and potentially misleading conclusions regarding participation rates, resource allocation, and policy effectiveness. Ensuring a standardized and equitable approach in the statistical treatment of all sports is crucial for obtaining accurate and reliable data. This, in turn, will inform better decision-making processes, promote fair resource distribution, and support the development of comprehensive strategies to enhance physical activity across all segments of the population.

The Austrian National Olympic Committee (NOC) primarily has a nomination function, focusing on the nomination procedures, administrative tasks, and sending athletes to the Olympic Games. According to Barth et al. (24), the sports ecosystem could be described as a “production network,” referring to highly decentralized and autonomous actors entitled to individually develop their own policies in respect to high-performance sports. In an operational sense, this is evident in the case of the national sport federations, as they exercise a dominant role in particular sports, especially in setting regulatory frameworks, including sports-specific rules. Though public and private sectors in Austrian sports governance operate with distinct responsibilities, they are highly interconnected. Despite high political and organizational autonomy, the National Sport Federation (NSF)'s operations primarily depend on public funds, contributing to the limited financial autonomy (38).

## Subnational (federal) competences in sport

Federal state laws naturally take precedence when a lack of legislative competence at the federal level exists, while the federal government can only engage in the realm of sports through forms of private law in general (63). The nine Austrian federal states make use of their legislative competences in the area of (sovereign) sports law under a variety of state laws. The federal states have passed state sport laws as part of this general competence. However, these state sport laws primarily include organizational law, sports funding law, and administrative matters. In addition, civil law, social security law, and liability law content also emerge in isolated cases.

On the federal state level, the following federal state sport acts form the legal basis for the respective federal state sport organizations: Burgenland Sport Promotion Act (BLGBl. 2004/26), Carinthia Sport Act 1997 (KLGBl 99), Lower Austria Sport Act 1997 (NÖLGBl 5710), Upper Austria Sport Act (OÖLGBl 1997/93), Salzburg State Sport Act 1988 (SLGBl 98), Styria State Sport Act 1988 (StLGBl 67), Tyrol Sport Promotion Act (TLGBl 2006/97), Vorarlberg Sports Act (VLGBl 1972/15), and Vienna State Sport Act (WLGBl 1972/17). Following Article 15 B-VG, competences in legislation and enforcement in sports matters are the responsibility of the nine federal states, enabling them to decide on sports laws within their sphere of influence, i.e., a particular federal state whose federal state parliament has decided to enforce it. Given the national relevance of sports and the BSFG (2017), provincial governments of the federal states are entrusted with sports matters and, therefore, are considered relevant state institutions. In addition, Austria's municipalities are important system partners, as they act as sponsors for sport clubs and providers of sport infrastructure on a local level (64).

The federal state sport organizations are—from a legal perspective—public law corporations with the predominant purpose of engaging in and promoting sport while maintaining their independence, representing and promoting the concerns and interests of sport, and advising the authorities of the state on all matters related to sport. The following example should give a better understanding of the decentralized governance model of sport in Austria and the development sports-specific policies with respect to constitutional jurisdictions.

## Federal state sport law in Styria

Established in 1988 and renewed in 2015, the Styria sport law puts sport under the umbrella of “culture, Europe, sport” in section 9 of the Styria federal state division. The State Sport Organization (of Styria) is a public corporation headquartered in Graz and is subject to oversight by the regional government with the main task of representing and promoting the interests and concerns of sports in Styria (65). The Department of Sport serves as a platform, interface, and administrative office, managing subsidies, legal matters, and the agendas of the Federal State Sport Center and the State Sport Organization. The department also oversees mountain and ski guiding and ski school operations.

Based on the State Law Gazette (LGBl.), the official journal of federal state legislation, Nr. 53/2015 § 5 (1) (38), the Styrian State Sport Organization was formed from the existing associations in Styria, whose primary or predominant purpose was the promotion of sports (professional associations, umbrella organizations, and their affiliated sports clubs). Associations not falling under the provision of § 1 but deemed significant for sports can be admitted as extraordinary members. The officials of the State Sport Organization carry out their duties on a voluntary basis (Styrian state government, 2015). According to § 5 (4), the bodies of the State Sport Organization are (1) the State Sports Council, (2) the State Sports Expert Council, and (3) the committees. The duties of the State Sport Council are outlined in § 7 of the Styrian Federal state sport law, including advising the regional government on all matters related to sports and sport promotion; providing recommendations on all issues associated with sport; representing the general interests of sport and those of the sport clubs, umbrella organizations, and specialty associations affiliated with the State Sport Organization; providing opinions on drafts of regional laws and regulations issued by the regional government concerning sport interests; decision-making regarding the rules of procedure for the State Sport Council and the State Sport Expert Council; drafting the budget for the State Sport Organization; and approving the annual financial statements, among others.

The State Sport Expert Council's role is to advise and support the regional government and the State Sport Council on all sports-specific matters, representing the interests of the sports-specific associations and contributing accordingly within the respective committees. The State Sport Expert Council consists of representatives from the sport associations. Each specialty association appoints one representative to the State Sport Expert Council (38). The State Sport Expert Council establishes permanent and optional committees to address specific tasks. There are permanent committees for recreational sport, competitive sport, infrastructure, education and training, and school and sport. The chairs of the committees are appointed by the State Sport Council. The number of members and the creation of rules and procedures for the committees also lie within the responsibilities of the State Sport Expert Council (38).

As outlined in Article 1 of the Styrian State Sport Law 2015, the goals are enabling physical activity and self-determined use of sport facilities for every individual, achieving international sporting success while considering measures against doping, securing the network of clubs and associations, empowering women, and promoting sport among girls. The outlined objectives are to be pursued by actively incorporating the diverse needs of individuals in Styria, ensuring equal participation in sports for everyone, regardless of age, gender, social or ethnic background, disabilities, beliefs, or sexual orientation.

Financial resources to ensure the fulfillment of the tasks of the State Sport Organization are primarily obtained through events organized by the State Sport Organization and through voluntary allocation of funds from other sports events; from revenues generated by the assets of the State Sport Organization, such as income from the rental of sport fields and facilities; through donations, legacies, collections, and other contributions; and via possible contributions and surcharges on the admission fees for sports events agreed upon by the State Sport Council and member clubs and grants provided by the state (65, § 10). As a public corporation, the federal state sports organizations are composed of sport specialty associations and umbrella organizations while preserving their individual characteristics and autonomy. In accordance with the Styrian federal state law, the Styrian Sport Strategy 2025 aims to get more people in Styria physically active (66). Divided into five chapters, the short and general document was developed in close collaboration with the organized sports associations in Austria. The Styrian capital city, Graz, is one of two federal state capital cities in the whole of Austria to have a sport strategy. Following major investments in sports infrastructure, the Sport Strategy Graz 2030 was developed with six overarching goals, building on the “active city” concept while referring to the UN Sustainable Development Goals and aiming to offer all people in the city of Graz the opportunity to participate in sports (67).

From the governmental perspective, sport is mentioned in the current government program as a subchapter, highlighting the volunteer sport sector, the importance of grassroot sports, and the development of the youth sport sector together with infrastructure. Special attention is given to the establishment of more ski schools and funding for swimming courses (68). The government program of the city of Graz (2021–2026) dedicates a subchapter to sport, underlining the relevance of elite sport and the further development of the grassroot sports level (69).

## Differences in federal state sports laws

The nine Austrian federal states execute their legislative competence within the framework of a large number of federal state laws (in the area of sovereign sport law). The state sport laws, as part of general competences, primarily include organizational law, sports funding law, and administrative matters. In addition, civil law, social security law, and liability law emerge in isolated cases. Due to the non-unified legislation, some federal states summarize their entire sports law, while others prefer the path of thematic fragmentation into, for example, sports, mountain guide, or ski school laws. While some federal states state that “sport in all its manifestations” should be supported and highlight the importance of sports in people's lives and society (§1 Para. 1 Lower Austrian Sport Act, §1 Para. 1 Z1–2 Upper Austria Sport Act 2019, §1 Paragraph 1 lit.a Tyrolean Sport Act 2006) and the relevance that sports have in maintaining health, in moral and physical education, and for international understanding (§1 par 2 of the Lower Austria Sport Act; §1 Paragraph 1 Salzburg Sport Act 2018), only one (out of nine) federal state sport law includes a definition of sport (Vorarlberg Sport Act, Section 1 Paragraph 2 of the Sport Act).

Special attention is paid in the Vorarlberg Sport Act (2022) (51) to the provisions regarding safety in sport practice, whereas the alpine police (Ger.: “*Alpinpolizei*”) was legally established, giving them the right/permission to report violations of recognized safety rules in skiing as administrative offenses (69, §6 and § 14). Moreover, seven of the nine federal states (except Vienna and Burgenland) have a separate ski school law. They were primarily based on monopoly approval, which was declared unconstitutional by the Constitutional Court (VfGH) in 1989 (61).

Sport funding also has some specific support measures in certain federal states. In Lower Austria, the broadcast tax, collected along with the broadcasting fee, has dedicated donations for sport funding (70). In Vienna, there is the “sport promotion contribution” (Ger.: “*Sportförderungsbeitrag*”)^6^ regulated in the Vienna State Law Gazette No. 22/2012, collected if the event had a (predominantly) sporting character, usually 10% of the entrance fee (excluding sales taxes). In exceptional cases, it can be reduced to 5% (71). Sport promotion in the federal states is carried out to a large extent within the framework of tourism promotion. Some federal states (Lower Austria, Upper Austria, Styria, and Vienna) have legal provisions for the protection of certain sports facilities. According to the Styria Sports Facilities Protection Act 1991 (72), the closure of certain sports facilities is only permitted with the consent of the municipality.

## Funding mechanism of sports

Sport funding in Austria has a long-standing tradition and is supported by the federal government, the federal states, municipalities, and from the revenue of the Austrian Lotteries. At the beginning of the Second Republic, there were limited financial resources available for sport from both the public sector and the economy. After some failed trials, the Sport Betting Regulation was enacted in 1949 (73). Sports betting regulations in Austria are the responsibility of the federal states.

From the sports perspective, the most important aspect of the Federal Gambling Act is “Toto.” The federal government makes an annual amount of 80 million Euros for sports promotion available from the license fee for Toto, according to Article 14 (GSpG, Article 20) (47).

The BSFG (81) built the legal basis for the federal funding of sport together with the GSpG. The Federal Sport Promotion Act 2017 established the Bundes-Sport GmbH (BSG) (Federal State Ltd.) as a subsidiary funding agency, 100% owned by the federal state. From 2023, the annual Special Sport Promotion was increased by 50%, from 80 to 120 million Euros for the first time since 2011 (73, § 20). As the funding agency, the goals of the BSG are outlined in Article 2 of the Federal Sports Promotion Act in accordance with the societal significance of sport in Austria, supporting the way toward a more professional structure of the sport sector in Austria. The focus areas are the Olympic Games and the support of athletes winning medals, supporting the advancement of competitive and performance sport as the foundation for elite sport. Furthermore, the enhancement of sport infrastructure, the encouragement of increased physical activity and sport participation for better health, and the inclusion of different groups are mentioned. The Federal Sport Promotion Act provides detailed criteria (by means of a criteria catalogue) for the transparent annual distribution of funds in accordance with § 20 of the Federal Gambling Act (38). Other funds are provided for in the Federal Finance Act for the promotion of projects of nationwide significance. These include special funds allocated to special tasks in sport (min. 1.11 million Euros), promotion of gender equality (min. 200,000 Euro), athlete-specific elite sports support provided to federal sports federations (min. 7 million Euros), and the promotion of institutions of national importance in sport, particularly in elite youth sport, sport science, and dual education (min. 4 million Euros) (74). At least 6.5 million Euros is set aside for subsidies to the Federal Sport Facilities Company Limited Liability in accordance with §§ 5, 10 of the Federal Sport Facilities Company Act, and funds for financing participation in Olympic events, Paralympic events, and Special Olympics events are also provided. Every subcategory has dedicated minimum financial support, but funds solely going to the NOC are not sufficiently detailed. This seems vague because the main duties and tasks of the NOC are the support of the participation of athletes in Olympic events, Paralympic events, and Special Olympics events (75). According to its significance, football receives different funding. Annually, 6.5 million euros is allocated to football, supplemented by 23.5% of the total funds reserved for the additional support of nationwide organizations with specialized responsibilities in the field of sports, in accordance with Article 5 of the BSFG.

The allocation of federal sports funding is distributed as follows: 50% for elite and high-performance sports, 45% for recreational sports, and 5% for the promotion of nationwide organizations with special responsibilities in sports, in accordance with Article 5 of the BSFG.

## Contemporary development trends

To date, Austria does not have a Professional Sport Act. The Austrian People's Party (ÖVP) and the Greens are proposing the development of a Professional Sport Act to improve the conditions for sports-specific professions in labor, tax, and social security law and establish legally secure conditions. Seasonal athletes, for example, are currently neglected as they are required to register with the Public Employment Service (AMS) outside the season. The establishment of a Professional Sport Act is also one of the points of the current government program (62). The proposed act is currently under revision.

Austria does not have a specific sport strategy or a national sport diplomacy strategy. The latter is of particular importance as the bulk of Austrian sport diplomatic efforts are carried out in the framework of bilateral and international cooperation through agreements and memoranda of understanding, the support sport-related initiatives in the UN Human Rights Council and the UN General Assembly, and the establishment of a working group on Sport and Human Rights (76). Austria is also part of the “*Conseil International du Sport Militaire*” and the “*Union Sportive Internationale des Polices*.” Sport also has a role in *ReFocus Austria*, a global economic initiative that was launched in September 2021 as part of the federal government's plan for economic reconstruction during and after the pandemic (77). In 2015, the Working Group on Sport and Human Rights was set up by the Ministry of Sport (78). In 2022, the Federal Ministry for Arts, Culture, the Civil Service and Sport, representing the Austrian Government, became the newest member of the multi-stakeholder Advisory Council of the Centre for Sport and Human Rights (79). In previous years, mega sporting events have been Austria's way of contributing to international understanding and the promotion of social and cultural values, hosting two Winter Olympics in Innsbruck in 1964 and 1976. In 2008, Austria hosted UEFA Euro 2008 together with Switzerland, and four years later, Innsbruck again hosted the Winter YOG. Since then, besides FIS Ski World Cup races, the Formula 1 Grand Prix in Salzburg, and the FIVB Beachvolleyball Grand Slam in Vienna, mega sporting events have not been on the agenda of Austria, removing the possibility of increasing the country's international prestige. Despite limited academic interest, this study provides a solid starting point for follow-up or in-depth research on political and policy aspects in Austrian sport. Moreover, the authors suggest focusing on researching the funding, organizational structure, and governance of the sports movement in Austria.

The repeated restructuring of the Ministry of Sport suggests that while sport are recognized as a relevant policy area, they have not been granted consistent institutional importance within the broader governmental framework. Addressing this issue may require a more strategic approach, ensuring that sport governance is anchored within a stable institutional setting that allows for long-term planning and sustainable policy development.

This research lays the basis for further groundwork on sport governance and policy and marks a significant contribution to the broader discourse by offering a comprehensive analysis of Austria's sport system, with particular emphasis on its historical evolution, regulatory frameworks, and key stakeholders. By examining the intersections of legal, institutional, and socio-political factors within the Austrian context, the study provides valuable insights that can inform comparative analyses with other European and global sport systems. This deeper understanding of the fundamental components and underlying mechanisms of the policymaking process can help practitioners to more effectively utilize sports as a tool for social engagement (36). Moreover, the identification of emerging trends and the exploration of innovative policy approaches contribute to the ongoing development of sports governance practices, providing a foundation for future policy reforms not only within Austria but also for broader international sport policy discussions.

The paper reveals crucial gaps in the academic literature on emerging trends in sport, including innovations in governance, digitalization, and inclusivity, reflecting the lack of interest in Austrian higher education for sport social sciences. This article discloses the requirement for many more academic papers associated with sport governance. Considering the recent committee elections of Sport Austria, gender equality within Austrian leadership structures appears to remain a distant goal. Currently, only 12% of committee positions are occupied by women, raising questions about the extent to which Sport Austria's initiatives—many of which aim to promote gender equality—are effectively implemented (80). This disparity suggests that the organization may face challenges in ensuring the full impact of its policies, potentially undermining the very objectives it seeks to advance in the realm of gender equality within the sports sector.

As current efforts to promote inclusivity still lack female representation, it is suggested that future research focuses on gender equality in Austrian sport leadership structures, potentially considering gender quotas as a possible solution to accelerate progress and ensure that women are adequately represented in decision-making bodies within the sport sector.

## Data Availability

The original contributions presented in the study are included in the article/Supplementary Material, further inquiries can be directed to the corresponding author.

## Footnotes

^1^European Youth Olympic Festival.

^2^Youth Olympic Games.

^3^International Children Games.

^4^At the time Federal Ministry for Art, Culture, Public Services and Sport.

^5^Nationaler Aktionsplan.

^6^It was called *Sportgroschen* until 2002. The name changed to “sports promotion contribution” (Ger.: “*Sportförderungsbeitragsgesetz*”).
